# Thioridazine: A Non-Antibiotic Drug Highly Effective, in Combination with First Line Anti-Tuberculosis Drugs, against Any Form of Antibiotic Resistance of *Mycobacterium tuberculosis* Due to Its Multi-Mechanisms of Action

**DOI:** 10.3390/antibiotics6010003

**Published:** 2017-01-14

**Authors:** Leonard Amaral, Miguel Viveiros

**Affiliations:** 1 Insititute of Hygiene and Tropical Medicine, Universidade Nova de Lisboa, Lisbon 1349-008, Portugal; 2 Institute of Medical Microbiology and Immunobiology, University of Szeged, Szeged 6720, Hungary; 3 Unidade de Microbiologia Médica, Global Health and Tropical Medicine, Instituto de Higiene e Medicina Tropical, Universidade Nova de Lisboa, Lisbon 1349-008, Portugal; MViveiros@ihmt.unl.pt

**Keywords:** antibiotic resistant pulmonary tuberculosis, thioridazine, in vitro activity, ex vivo activity, in vivo activity (murine model and human trails), the non-killing macrophage, enhancement of intracellular killing of Mtb by thioridazine, mechanism of actions of thioridazine

## Abstract

This review presents the evidence that supports the use of thioridazine (TZ) for the therapy of a pulmonary tuberculosis infection regardless of its antibiotic resistance status. The evidence consists of in vitro and ex vivo assays that demonstrate the activity of TZ against all encountered *Mycobacterium tuberculosis* (Mtb) regardless of its antibiotic resistance phenotype, as well as in vivo as a therapy for mice infected with multi-drug resistant strains of Mtb, or for human subjects infected with extensively drug resistant (XDR) Mtb. The mechanisms of action by which TZ brings about successful therapeutic outcomes are presented in detail.

## 1. Introduction

The resurgence of tuberculosis during the 1980s was followed in the 1990s in New York City by a dramatic increase in the rate of pulmonary tuberculosis (TB) infections accompanied with emerging levels of resistance to the first line anti-tuberculosis (anti-TB) drugs isoniazid and rifampicin—termed multi-drug resistant tuberculosis (MDR-TB) [1]. Resistance to these drugs plus resistance to the second line drugs, amikacin, kanamycin, capreomycin and fluoroquinolones—termed extensively drug resistant tuberculosis (XDR-TB)—was soon noted in various parts of the globe [2]. More recently, resistance to all anti-tuberculosis drugs—termed totally drug resistant tuberculosis (TDR-TB) was first noted in Italy in 2006 and later reported in Iran, India and South Africa [3]. Although the global acquisition of tuberculosis infections has decreased by 1.5% per year [4,5], the progression of increased resistance of *Mycobacterium tuberculosis* (Mtb), as a consequence of prolonged and problematic therapeutic regimens, threatens the progress that has been made since 1990 in the control and prevention of TB [5]. Although the efficacy of the repurposed and newly recommended antibiotic for resistant forms of Mtb—Linezolid—has been recently evaluated from data provided by 23 studies in 14 different countries, involving more than 500 patients, suggests an overall success rate of 77% [6], the drug is notorious for producing a plethora of serious side effects such as neuropathy and hematological disorders [7]. Other newly approved drugs for MDR and XDR-TB therapeutics—bedaquiline and delamalid—are following the same path with their recognized efficacy against resistant forms of TB being threatened by their market cost and cumulative reports of side effects and lack of safety [8]. Despite the occurrence of these side effects, in the absence of better forms of effective therapeutic regimens, the World Health Organization (WHO) continues to recommend the use of these extremely costly drugs for therapy of MDR and XDR-TB [4,6,8]. But is there an inexpensive drug, in comparison to the cost of these drugs, that has been extensively studied and has been safely used for the therapy of psychosis for over 50 years producing no serious side effects if the patient are monitored properly? The answer resides in thioridazine!

It is the purpose of this review to present all of the evidence, much of it confirmed by many groups around the world, which strongly supports that use of thioridazine (TZ) in combination with antibiotics to which the Mtb isolate was previously resistant for therapy of MDR, XDR and most probably, TDR.

## 2. *Mycobacterium tuberculosis* and Phenothiazines: Chlorpromazine, Thioridazine, in Vitro Activities

**Figure 1:**
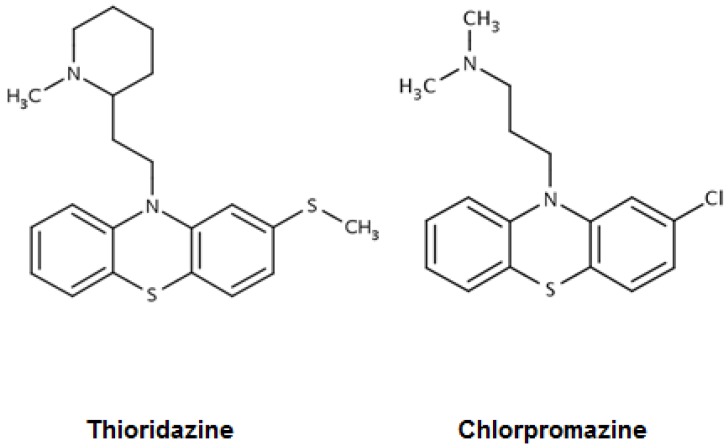
Structures of the phenothiazines thioridazine and chlorpromazine [9].

Phenothiazines are heterocyclic compounds. The first such compound was synthesized by Bernthsen in 1883 via the reaction of diphenylamine with sulfur. Methylene blue (MB) is a phenothiazine that was synthesized from a phenothiazine derivative by Heinrich August Bernthsen in 1883. Soon thereafter, the chemist Paul Erhlich used the MB dye for staining live cells and found that it could reduce movement of microorganisms [9]. This observation spurred experiments with humans that showed that the dye could render the subject sedated and was effective in the treatment of schizophrenia [10]. These discoveries led to the synthesis of chlorpromazine (CPZ) by Paul Charpentier in 1950, introduced by Rhone Poulenc as the first true neuroleptic in 1957 [10] (Figure 1).

Because of its worldwide use, anecdotal observations suggested that it had antimycobacterial properties [11]. By 1977, the in vitro antimycobacterial properties of CPZ were clearly shown [12] and confirmed 10 years later [13]. As a consequence of the emergence of a pulmonary tuberculosis epidemic in New York City during the late 1980s, and later the large percentage of MDR-TB coupled with the absence of new and effective anti-TB drugs at that time, the search for new anti-TB drugs began. The observations that CPZ had potential anti-TB activity spurred the study demonstrating that the in vitro concentrations of CPZ needed to inhibit the replication of Mtb could be exceeded and safely achieved in the CPZ treated patient, and clinically relevant concentrations ex vivo could effectively promote the killing of phagocytosed Mtb [14]. Regrettably, CPZ is also a drug that causes very serious side effects [15]. Thioridazine (TZ) is an equally effective neuroleptic phenothiazine. It produces significantly fewer side effects when used with moderation and maintaining an evaluation of the patient for underlying cardiopathy. TZ is therapeutically safe, as proven by the 60 plus years it has been in use, and is still widely used today in many countries to control psychosis. Consequently, TZ was examined for in vitro activity against antibiotic susceptible and antibiotic resistant isolates of Mtb and compared to the activity of CPZ against the same strains [16]. The MICs in vitro for CPZ and TZ were calculated as ranging between 4 and 32 μg/mL, depending on the system and the antibiotic resistance status of the tested strain, and they were equally effective [17]. For the *M. tuberculosis* H37Rv fully antibiotic susceptible reference strain this range was determined, by many authors, to be 8–15 µg/mL depending on the system (Table 1).

**Figure 2:**
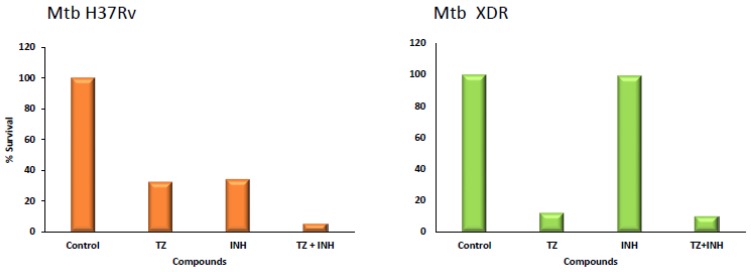
Killing effect of thioridazine over three days of infection. Effect of thioridazine on the intracellular survival of *M. tuberculosis* (Mtb) H37Rv and an XDR Mtb strain within human monocyte-derived macrophages at day three post infection. Isoniazid (INH) was tested at 0.1 µg/mL, and thioridazine (TZ) at 2.5 µg/mL. Data are presented as a mean of the percentage of the survival (adapted from [26]).

Both CPZ and TZ had similar activity against strains susceptible to isoniazid (INH) and rifampicin (RIF) as well as to strains resistant to these antibiotics and as many as five other antibiotics. The in vitro effects of TZ [20,27,28] as well its derivatives, have since been repeatedly confirmed [23,29]. However, the minimal inhibitory concentrations that completely inhibited the replication of Mtb in vitro employed in all of the cited studies exceeded many fold that which can be safely achieved clinically (ca. 0.5 mg/L of plasma in a patient chronically treated with TZ). Because CPZ had been shown to reduce the resistance of a number of pathogenic bacterial species to antibiotics [30], presumably by interacting with the cell wall of the bacterium [31], the effect of TZ on the resistance of isolates of Mtb to antibiotics was also evaluated (Figure 2). Briefly, although all of the phenothiazines tested were able to reduce the resistance to first line anti-TB drugs, the very mild neuroleptic TZ demonstrated great effectiveness at concentrations that were clinically achievable and similar to those employed for the initial therapy of psychosis [32]. Although the mechanism by which TZ reduced antibiotic resistance of Mtb was not readily understood, studies in other groups of bacteria demonstrated that TZ reversed the resistance of *Escherichia coli* to tetracycline by inhibiting the over-expressed efflux pump of the bacterium that was responsible for its multi-drug resistant phenotype [33]. Consequently, a large number of clinical isolates of Mtb that were susceptible to isoniazid (INH) were induced to extremely high level resistance to INH; this resistance could be totally reversed with a small and clinically relevant concentration of TZ [34]. Further studies showed that TZ reversed resistance of Mtb that had been induced to high level resistance to INH via the interference with the over-expressed efflux pumps genes of the organism *mmpL7*, *p55*, *efpA*, *mmr*, *Rv1258c* and *Rv2459* [35] thereby confirming the previous observations on the role of efflux pumps in the multidrug resistance of the organism [34,36].

The effect of TZ on Mtb is not limited to the inhibition of efflux pumps. Studies by Dutta et al. show that, besides efflux pumps, the genes that code for essential proteins of the cell envelope are affected by TZ, albeit at concentrations that exceed the minimum inhibitory concentration of the drug [37]. Among the genes affected were those that encode efflux pumps that extrude antibiotics, oxido-reductases, enzymes involved in fatty acid metabolism and aerobic respiration, and genes that are co-expressed with the global SigmaB regulon, which are involved in the response to stress [38]. Other studies have confirmed these observations and have extended the understanding that TZ affects a large number of essential genes that code for proteins of the plasma membrane, many of which are involved in controlling essential energy production, active transport and permeability processes in response to antibiotic and oxidative stress stimuli [39]. In particular, several studies confirmed that TZ acts in mycobacterial respiratory chain components involved in ATP oxidative phosphorylation, namely, the type-II NADH-menaquinone oxidoreductase (NDH-2)—a key component of respiratory chain of Mtb—thus raising the hypothesis that this is the main molecular target of TZ and making it also effective against latent TB [40,41,42]. NDH-2 catalyzes the first reaction of the electron transfer chain of Mtb that leads to ATP oxidative phosphorylation. During this reaction, NDH-2 transfers two electrons from NADH to menaquinone, which is reduced to menaquinol form. Yano et al. have shown that the respiratory functions leading to de novo ATP synthesis and NADH regeneration might be the Achilles’ heel of hypoxic nonreplicating mycobacteria, making TZ an attractive drug with activity both against replicative and dormant mycobacteria [40,41,42]. This hypothesis has been confirmed by Sohaskey et al. who demonstrated that concentrations of TZ exceeding the MIC for actively replicating Mtb also inhibit/kill dormant Mtb, becoming a promising drug to control latent tuberculosis and shorten anti-TB drug regimens if used directly on the human macrophage [43,44]. However, the question of whether TZ can be clinically useful for inhibiting the replication of Mtb and simultaneously killing dormant Mtb remains doubtful unless science demonstrates that these effective in vitro concentrations can be achieved at the site of the pulmonary system where the infective organism normally resides, namely, the pulmonary macrophage. Because TZ is concentrated by cells such as macrophages that are rich in their lysosome content [45,46] to levels that theoretically are assumed to greatly exceed the concentration present in the medium (in fact never measured inside the macrophage, only measured in TZ-loaded culture lysates) [14,47], the noted effects of TZ on essential genes may take place in vivo.

## 3. Thioridazine and Its Effect on Intracellular *Mycobacterium tuberculosis*

To test the hypothesis above, and based upon the evidence that TZ was equal to CPZ with respect to its antimycobacterial properties in vitro (and the fact that CPZ was also very effective ex vivo), the rationale and the experiments performed by Crowle et al. [14] were repeated by Ordway et al. with CPZ and TZ against clinical strains of MDR Mtb [24], and later by Machado el al. against XDR Mtb, where TZ showed an excellent synergistic effect with first line drugs [26] (See Figure 1 as an example). In these works TZ was shown to enhance the killing of intracellular antibiotic susceptible and MDR/XDR Mtb by monocyte-derived human macrophages that have little killing action of their own at concentrations in the medium which are equivalent or lower than those present in the plasma of a thioridazine-treated psychotic patient (0.5 mg/L of plasma). These TZ ex vivo studies were extended to a large number of TZ derivatives, some of which revealed to be more effective than TZ and all of which expressed no toxicity at their effective ex vivo concentrations [29]. The same rationale and technical approach was further expanded from TZ and CPZ to other ion channel blockers such as verapamil, flupenthixol and haloperidol, with very successful results in enhancing the killing activity of the infected macrophage, regardless of the drug resistance profile of the infectious Mtb, with moderate and acceptable toxicities and excellent synergistic effects with first and second line anti-TB drugs [26].

The mechanism by which TZ promotes the killing of intracellular Mtb was at first opined to be the result of TZ being concentrated within the phagolysosome as predicted by Daniel and Wojcikowski [45,46] to a level compatible to its minimum bactericidal concentration of 60 mg/L. Although the concentrated effect in phagocytosed mycobacteria may take place, up to now this has never truly been demonstrated to occur in vivo. Controversial and disputed studies of Segal and associates [48,49] have also suggested another hypothesis which, if correct, could alter the form of therapy used against the MDR and XDR Mtb [24,50].

**Figure 3:**
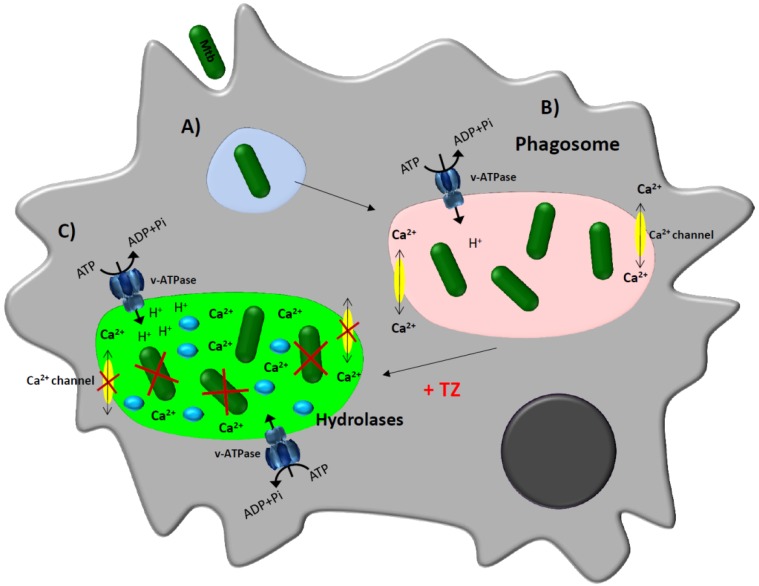
Schematic model proposed for the enhancement of the macrophage killing activity by thioridazine [26,50,62,69,71]. Infected macrophage. (**A**) The bacterium is recognized by receptors present on the plasma membrane of the macrophage and is internalized by invagination of the plasma membrane into a phagosome; (**B**) Once the phagosome is formed, the bacteria will manipulate the immune response, leading to the reduction of the availability of calcium within the phagosome, preventing the process of acidification needed for the activation of the hydrolases and the bacteria are thus not killed; (**C**) Treatment of infected-macrophages with Ca^2+^/ion channel blockers such as thioridazine (TZ) will increase the concentration of calcium into the cytoplasm and the transcription and activity of vacuolar proton (H+)-ATPases. This rise of protons causes the decrease of the pH in the phagolysosome, activating hydrolases that consequently kill the mycobacteria.

This hypothesis involves a series of stages that begin with: binding of the infecting Mtb organism to the receptor of the plasma membrane of the macrophage [51,52,53], followed with the invagination of the receptor-mycobacterium forming the phagosome which travels through the cytoplasm of the macrophage [54,55] and eventually fuses with a lysosome [56] to form the phagolysosome unit. The activation of dormant hydrolases (zymogen granules) requires a low pH [56] which is created by vesicular ATPases of the phagolysosome unit which are dependent upon the retention of ions [57]. Because the macrophage plasma membrane have their Ca^2+^ channels (L-type) inhibited in presence of TZ, this inhibition will result in a significant increase of Ca^2+^ from intracellular stores within macrophages. Accumulation of these ions in the cytoplasm of the macrophage causes an indirect acidification of the phagolysosome (schematic overview in Figure 3). Consequently, we considered the possibility that since TZ inhibits efflux pumps of bacteria [34,35,36] and also acts against efflux pumps of human cells [58,59,60], and phenothiazines in general inhibit Ca^2+^/ion channel transport [25,61], TZ may also inhibit the efflux of ions from the phagolysosomal unit leading to the indirect acidification of the compartment and the activation of hydrolytic enzymes. This possibility is supported by recent studies of Machado et al. [26,35] demonstrating that TZ promotes the acidification of the phagolysosomal unit by indirect inhibition of macrophage ion channels. The inhibition of these channels therefore activates the hydrolytic enzymes via the coupling of the vesicular ATPases and consequent killing of the entrapped Mtb organism. This hypothesis is further supported by separate research using another ion channel blocker, verapamil [62,63]. These studies not only provide support for the use of TZ for therapy of Mtb drug resistant infections by a non-antibiotic compound [64,65,66,67], but also introduce an alternative therapeutic strategy that targets the killing machinery of the pulmonary macrophage infected with Mtb [62,63,67,68,69,70]. Drugs that target mycobacteria will eventually cause the organism to become resistant via the development of mutations at the gene coding level of the antibiotic target, and the alternative form of therapy with TZ evades this mutagenic response and assists the still effective antibiotics against drug resistant forms of Mtb.

In Figure 3 a model of the putative mechanism of action of thioridazine inside the macrophage is depicted, combining all the contributions made so far to elucidate its remarkable enhancing activity of the macrophage killing activity [26,48,49,50].

## 4. Mono and Combinational Therapy with TZ: The Mouse and the Human

The question of whether the in vitro and ex vivo effects of TZ are reflected successfully in the murine model needed answering, and to this end, mono-TZ therapy of the Mtb infected mouse [72] as well as combination therapy with first line antibiotics in this model have both proven to be effective [73,74,75]. Nevertheless, because the results in the mouse model not always are reproduced in humans, the effectiveness of TZ-combination therapy needed to be investigated. To this end, TZ in combination with antibiotics to which the infective organism was initially resistant produced complete cures in 17 out of 18 XDR-TB patients in Argentina [76]. Mono-therapy of five terminal XDR-TB patients with TZ significantly improved their quality of life (elimination of night sweats, improved appetite, weight gain, reduction of disease-associated stress) and did contribute to a longer life span [71], but because TZ does not restore lost pulmonary tissue, the patients succumbed to the disease. Studies by Abbate et al. [70] and Udwadia et al. [77] showed that the use of TZ was safe with no significant effects on QT intervals or any other cardiac property as per the rigorous monitoring carried out in these trials.

## 5. Important Considerations for Therapy of MDR/XDR Mtb Patients with TZ in Combination with Antibiotics to Which the Infecting Organism Is Resistant

The initial response of bacteria to an antibiotic or noxious agent is to over-express its efflux pumps [26,33,34,35,78,79,80,81,82,83,84,85,86,87,88]. When the concentration of the agent is progressively increased, the genes that control and code for the efflux pumps of the responding organisms are progressively increased [33,34,35,85,87,88,89,90]. However, when the initial concentration of the antibiotic is maintained below its MIC during repeated passages, the bacterium responds with progressive increases in its efflux activity of the pre-existing pumps [26,35,90]. Eventually, the appearance of resistance to a large variety of unrelated antibiotics begins to occur with a progressive concomitant increase of transport activity ultimately leading to the MDR phenotype with basal (normal) levels of efflux activity [35,90]. These observations tend to explain why antibiotic resistance of an infecting bacterium continues to increase although the dosing of the patient remains unchanged [27,79,84]. Moreover, they also suggest that in order to define the antibiotic status of a clinical isolate from a patient who may be a suitable candidate for adjunct therapy with TZ, the antibiotic profile as well as the activity of the efflux pump system of the infecting organism should be determined. Whereas the determination of an antibiotic resistance panel is routine for a laboratory that performs diagnostic studies for a suspected pulmonary tuberculosis infection, there are at this time few laboratories that perform the needed assays that define the efflux pump status of the infecting Mtb isolate. Fortunately, there are methodologies that have been developed which are not difficult to perform by a routine tuberculosis laboratory that do not require expensive instrumentation. When this cost is compared to the huge cost associated with therapy of an MDR Mtb infection due to mutations or to an over-expressed efflux pump system, where therapy is expected to be highly problematic [91], the cost is indeed minor. Based upon the above, any patient who is considered to be a candidate for adjunct TZ therapy with antibiotics must first have a clinical isolate evaluated for susceptibility to first line antibiotics. The status of the efflux pump system and the ability of TZ to reverse its in vitro resistance to specific antibiotics of the panel must also be investigated before treatment [91]. In addition to these assays, it would be of great interest to determine the effect of TZ on the survival of the infecting isolate by the patient’s own macrophage. Given positive answers from the above assays (antibiotic susceptibility panel; defined efflux pump system; ability of TZ to reverse resistance to the antibiotic(s) for which the isolate was initially resistant; and, effective enhanced killing activity of the macrophage-trapped isolate), the patient may well be a good candidate for therapy with TZ as an adjunct to antibiotics whose initial resistance was due to an over-expressed efflux pump system [91]. During the time the clinical isolate is accordingly being investigated as suggested, the patient must be evaluated for any cardiopathy. It must be noted that the use of TZ is safe and the suggested protocol for dosing the patient is one that begins with a low level of 25 mg/day that is increased weekly to 50, 100 and 200 mg/day. This protocol has been shown not to reduce the QT interval (increased time between contractions of left and right ventricles) [92,93,94], a side effect repeatedly noted in MDR-TB patients treated with fluoroquinolones, bedaquiline or delamanid and a limitation factor for the use of new regimens including synthetic drugs [95]. However, approximately 6% of the Eastern European population has a mutation in the p450 cytochrome which reduces the metabolism of TZ, and consequently, the build-up of plasma TZ levels will result [93]. This build-up may be rapid and reach levels which are known to reduce the QT interval [94]. Consequently, the patient should be monitored for cardiac function prior to therapy with TZ in order to rule out any cardiopathy that may worsen with TZ dose, and monitoring should continue for the first week of therapy with TZ and periodically thereafter. It is important to note that TZ is safe to use for up to 1000 mg/day when introduced to the patient gradually [76,77,93,94], coupled to knowing the patient’s clinical history and performing cardiac monitoring as recommended. At this time, the time required for therapy leading to a negative TB culture and radiological evaluation consistent with cure is not known although as per Abbate et al. complete cures were achieved with XDR-TB patients within a few months of TZ adjunct therapy [76,77]. It may well be that full recovery of XDR TB patients takes place within a period of time commensurate with that routinely producing complete cures of the patient infected with antibiotic susceptible Mtb with daily doses that are far below those used for the therapy of a psychotic patient.

## 6. Costs Associated with the Care of an MDR-TB Patient

The average cost for hospitalization during the period from 2005 through 2007 for an MDR-TB patient in the USA was $81,000 per year and for the XDR-TB patient $285,000 (3.5 times than that for the MDR-TB) [96]. Regardless of this huge expenditure per patient, the mortality rate for MDR-TB in the USA is still significantly higher than that for antibiotic susceptible TB infections, and for XDR-TB significantly higher than for MDR infections, especially if the patient is co-infected with HIV or presents with AIDS [97]. Nevertheless, due to the development of a variety of clinical diagnostic programs, therapeutic monitoring such DOTS, and the wide introduction of rapid laboratory methods for the identification, isolation and susceptibility test to first line TB drugs, the frequency of TB infections susceptible and resistant to first line TB drugs has fallen dramatically [4,97]. In countries that are poor, the situation is totally reversed and the incidence of all forms of TB infections continues to rise rapidly [5,98]. Although it is not possible at this time to advance the status of TB control, therapy, etc. in these global regions, given the severity of increasing antibiotic resistance, it is reasonable that therapy of a clinical presentation of tuberculosis can be pursued without the luxury of what is present in wealthier countries. However, therapy with first line drugs is costly and if not properly administered leads to MDR, and progressively more resistant forms of TB. WHO has recommended linezolid to be included in the empirical protocols for the therapy of MDR/XDR-TB infections [4,5]. However, this drug has a very narrow therapeutic window and because the optimal dosing strategy that minimizes the substantial toxicity associated with prolonged use has not been determined [99,100], blind use of this drug is extremely expensive and problematic. Consequently, if far less expensive drugs such as those that make up the line of defense are available, and because TZ is safe when used as prescribed, and because the effective daily dose of TZ used by Abbate et al. was, via increments, limited to 200 mg/day [76], TZ in combination with first-line drugs may prove to be significantly effective for therapy of any form of tuberculosis. Given that TZ when concentrated by the phagolysosome will be effective against the efflux pumps that are responsible for MDR phenotype of the bacterium, and given that TZ may also reach a level in the phagolysosome compatible with that which is bactericidal in vitro, and coupled to the enhancement of killing by the macrophage housing the infective organism, the potential that TZ has for therapy of any TB pulmonary infection is significant and should continue to be further supported.

## 7. Conclusions

The body of results and evidences gathered so far, coming from many different contributions from different teams around the world, enable us to propose the following mechanism of action for thioridazine and other ion channel blockers in the bacteria: after entering the cell, the compounds will generate a cascade of events which starts with the inhibition of the respiratory chain complexes, though we cannot say at the present moment if the respiratory chain is a direct target. The inhibition of the bacterial respiratory chain will lead to dissipation of the membrane potential, reduction of ATP levels, efflux inhibition, oxidative stress, and increase in intracellular ion levels. On the host cell, treatment with these compounds results in phagosome acidification that synergizes with several components of the host immune response, such as lysosomal hydrolases, leading to bacterial growth restriction. Both effects cooperate and result in an enhanced killing activity that can be highly efficient when combined with antituberculosis drugs.

Promising examples of the future use of thioridazine in new short term therapeutic regimens against any form of antibiotic resistance of Mtb come from the recent studies that demonstrated the possibility of effectively using nanoparticles containing thioridazine and rifampicin for rapid tuberculosis treatment in vitro and in a zebrafish model [101,102]. The use of TZ as a therapeutic adjuvant for anti-TB therapy is currently being expanded in Argentina and India. Even the World Health Organization, who has not shown great interest in the repurposing of this narcoleptic drug for TB has recently considered thioridazine as a World Health Organization group 5 drug for multidrug-resistant tuberculosis treatment due to its efficacy and safety [103].

## Figures and Tables

**Table 1:** Minimum inhibitory concentrations of thioridazine for *M. tuberculosis* H37Rv reference strain according to the literature.

Culture Systems	TZ	References
MICs		
MB7H10	8–10 μg/mL	[18,19,20]
Broth microdilution	8–15 μg/mL	[21,22,23]
Bactec TB-460	15 μg/mL	[24,25]
Bactec MGIT 960	15 μg/mL	[26]
Cytotoxicity *		
IC^50^	13.78 μM	[23]

* Evaluated against human monocyte-derived macrophages after 72 h of incubation at 37°C, 5% CO_2_.
